# A Delphi Study to Detect Deficiencies and Propose Actions in Real Life Treatment of Neovascular Age-Related Macular Degeneration

**DOI:** 10.1155/2014/595132

**Published:** 2014-12-21

**Authors:** Alfredo García-Layana, Luis Arias, Marta S. Figueroa, Javier Araiz, José María Ruiz-Moreno, José García-Arumí, Francisco Gómez-Ulla, María Isabel López-Gálvez, Francisco Cabrera-López, José Manuel García-Campos, Jordi Monés, Enrique Cervera, Felix Armadá, Roberto Gallego-Pinazo, Antonio Piñero-Bustamante, Miguel Angel Serrano-Garcia

**Affiliations:** ^1^Clínica Universidad de Navarra, Avenida de Pío XII 36, 31008 Pamplona, Spain; ^2^Hospital de Bellvitge, C/Feixa Llargasn, L'Hospitalet de Llobregat, 08907 Barcelona, Spain; ^3^Hospital Universitario Ramon y Cajal, Carretera de Colmenar km 9, 28034 Madrid, Spain; ^4^Vissum Madrid, Santa Hortensia 58, 28002 Madrid, Spain; ^5^Hospital de San Eloy, Avenida Antonio Miranda 5, 48902 Baracaldo, Spain; ^6^Hospital Universitario de Albacete, Avenida de Almansa, s/n, 02006 Albacete, Spain; ^7^Hospital VallD'Hebron, Passeig de la Valld'Hebron 119-129, 08035 Barcelona, Spain; ^8^Instituto Oftalmológico Gómez-Ulla, Calle Maruja Mallo 3, 15706 Santiago de Compostela, Spain; ^9^IOBA, Hospital Clínico Universitario de Valladolid, Paseo de Belén 17, 47011 Valladolid, Spain; ^10^Complejo Hospitalario Universitario Insular Materno Infantil de Gran Canaria, Avenida Marítima del Sur, s/n, 35016 Las Palmas de Gran Canaria, Spain; ^11^Hospital Universitario Virgen de la Victoria, Campus de Teatinos, s/n, 29010 Málaga, Spain; ^12^Institut de la Macula i de la Retina, Carrer de Vilana 12, 08022 Barcelona, Spain; ^13^Hospital General de Valencia, Avenida Tres Cruces 2, 46014 Valencia, Spain; ^14^Hospital la Paz, Paseo de la Castellana 261, 28046 Madrid, Spain; ^15^Hospital la Fe, Valencia, Avenida de Fernando Abril Martorell 106, 46026 Valencia, Spain; ^16^Hospital Universitario de Valme, Avenida de Bellavista, s/n, 41014 Sevilla, Spain; ^17^Hospital Universitario Nuestra Señora de la Candelaria, Carretera del Rosario 145, 38010 Santa Cruz de Tenerife, Spain

## Abstract

*Purpose*. Spanish retina specialists were surveyed in order to propose actions to decrease deficiencies in real-life neovascular age macular degeneration treatment (nv-AMD).* Methods*. One hundred experts, members of the Spanish Vitreoretinal Society (SERV), were invited to complete an online survey of 52 statements about nv-AMD management with a modified Delphi methodology. Four rounds were performed using a 5-point Linkert scale. Recommendations were developed after analyzing the differences between the results and the SERV guidelines recommendations.* Results*. Eighty-seven specialists completed all the Delphi rounds. Once major potential deficiencies in real-life nv-AMD treatment were identified, 15 recommendations were developed with a high level of agreement. Consensus statements to reduce the burden of the disease included the use of treat and extend regimen and to reduce the amount of diagnostic tests during the loading phase and training technical staff to perform these tests and reduce the time between relapse detection and reinjection, as well as establishing patient referral protocols to outside general ophthalmology clinics.* Conclusion*. The level of agreement with the final recommendations for nv-AMD treatment among Spanish retinal specialist was high indicating that some actions could be applied in order to reduce the deficiencies in real-life nv-AMD treatment.

## 1. Introduction

Age-related macular degeneration (AMD) is the most common cause of blindness in the Western world, and given its chronic and progressive course altogether with the fact that AMD may require lifelong observation and therapy, it has become a major socioeconomic issue as the proportion of the aged population is increasing exponentially [1]. The beneficial effects of intravitreal injections of ranibizumab (Lucentis, Genentech, Inc., South San Francisco, CA, and Novartis, Basel, Switzerland) and aflibercept in the treatment of neovascular AMD (nv-AMD) have been widely evidenced showing significant improvements in visual acuity and quality of life [2, 3]. The standard of care for nv-AMD was established on basis of the results obtained in the pivotal randomized clinical trials with fixed monthly injections of ranibizumab [2], albeit a significant burden for patients, physicians, and health services. Retinal specialists developed further modifications to this monthly regime. For instance, an individualized dosing strategy through a monthly follow-up with the decision to treat based on optical coherence tomography (OCT) findings and visual acuity changes arose as a potential alternative. Several reports have shown that intravitreal antiVEGF therapy with ranibizumab or with bevacizumab is effective in maintaining or improving visual acuity on this* “as-needed”* basis, so called* pro re nata* (PRN) strategy; [4–6] furthermore, ranibizumab administered on an as-needed basis was a more cost-effective strategy compared to fixed monthly treatments with respect to cost per QALY gained [7].

More recently it has been reported that, after extended follow-up periods, most patients exhibit persistent exudation of the choroidal neovascularization (CNV) with moderate vision loss with this PRN therapeutic approach [8]. The HORIZON study demonstrated that after 3 and 4 years of follow-up of patients with neovascular AMD, an incremental decline of the VA gains seen during the first 2 years of the studies took place after patients were switched to a PRN regimen, suggesting that switching from a strict treatment regimen to a less frequent investigator-determined PRN dosing was paralleled by signs of disease destabilization [9].

A variety of publications of cohorts of patients with nv-AMD managed with a PRN strategy have been reported: a Swedish study showed an increase of 1 letter after 12 months, a Spanish showed an increase of 1.3 letters after 12 months, and a German study reported a stabilization of vision after 24 months [10–12].

This significant worsening of visual acuity after 12–24 months of follow-up may be related to a decrease in the number of follow-up visits and intravitreal drug injections when compared with the clinical trials. For instance, a Danish study with a mean number of injections of 8.7 in four years found a significant decrease in vision through that period [8].

Individualized treatment regimens impose a considerable burden for the patient, and good results might only be achieved if patients were able and willing to accept these monthly visits. However, the retinal physicians' nonadherence to the nv-AMD treatment guidelines and the health systems organization may preclude an optimum treatment in some patients, making the translation of the clinical trial results to real life outcomes impossible. The decline in quality of life and increased need of daily living assistance after long follow-up of patients with nv-AMD substantiates the need for detecting deficiencies in real-life treatment in order to prevent vision loss and progression to blindness.

The purpose of this study was to perform a Delphi study with Spanish experts in nv-AMD treatment to identify major potential problems in its management and to propose actions that may improve the final outcomes of these patients.

## 2. Methods

### 2.1. Study Design

A national survey was performed using a modified* Delphi process* [13]. The* Delphi process* is an anonymous group facilitation technique that seeks to obtain consensus on the opinions of experts through a series of structured questionnaires, called rounds. After each one of these rounds the results are summarized and presented to the participants for possible reconsideration of their opinion. The anonymous nature of the Delphi technique ensures that a single individual cannot dominate the consensus formation and all participants are equally able to change their opinion in the course of the process [14–16].

In the present study a modified Delphi method was used, with the items to be surveyed previously selected by a general coordinator and discussed with an expert panel instead of provided by the participants.

### 2.2. Selection of Survey Targets

Retinal experts were identified representing proportionally all the regions in Spain according to the following selection criteria: (1) Spanish Vitreoretinal Society (SERV) members; (2) active nv-AMD referral specialist; (3) involvement in postgraduate training.

As there is no formal consensus about the number of experts to be required for a Delphi study, a total of 100 retinal physicians were distributed according to the number of people aged over 50 years who live in the 52 Spanish provinces; sixteen leading SERV members (expert panel) identified these 100 experts based on the criteria afore mentioned.

The key issues about nv-AMD treatment to be surveyed were identified by the study coordinator (Alfredo García-Layana) and discussed with the expert panel based on the nv-AMD SERV guidelines [17].

### 2.3. First Delphi Round

An anonymized questionnaire was e-mailed to the 100 retinal physicians selected, including the guidelines to rate how often they performed several tasks or agree/disagree with different statements. They were requested to submit an email to the study coordinator with any relevant comment about the questionnaire and were encouraged to suggest new items that should be considered for further assessments. Each item was rated into a four-step scale provided with response anchors (1 = almost never; 2 = sometimes; 3 = frequently; 4 = almost always).

### 2.4. Second Delphi Round

The ratings resulting from the first Delphi round were analyzed and the scores distribution was presented to the expert panel revised in a presentational meeting. All the submitted comments were addressed and ambiguous questions were reformulated. The questions remained unchanged but clarification was allowed. All new proposed elements were discussed and added next to the questions describing similar items, if they were considered relevant by the advisory panel. The study coordinator submitted the new survey to the entire participant pool that responded to the first round. Each one of the previous questions was provided with an anonymous summary of the expert's opinion from the previous round. The study participants were encouraged to revise their earlier answers in light of the replies of other members of their panel. No elements were excluded between the first and second rounds.

### 2.5. The Third and Fourth Delphi Rounds

The final content of the assessment instrument was based on the consensus achieved after the second Delphi round. Differences between the results of the survey and the recommendations of the nv-AMD SERV guidelines [17] were analyzed. Thereafter, a third round with recommendations developed by the study coordinator was resubmitted with the same strategy used for the first round. Their opinions were measured using a five-point Linkert scale ranging from strongly-agree to strongly disagree. A fourth Delphi round was performed to review their earlier answers in light of the replies of other members.

A predefined consensus level is an indicator good quality and best practice guidelines suggest that criteria for consensus should be defined in advance [18]. So, consensus was predefined as more than 70% of the experts supporting an element answering the item with “strongly agree” or “agree” [19–21].

## 3. Results

One hundred retina specialists were selected from a total of 537 SERV members. Eighty-seven completed the first Delphi round of answers, and 85 completed the second round. Sixty-nine of them (81%) had more than 10 years of experience treating vitreoretinal diseases. Forty-eight (56%) work both in National Health Service (NHS) and private facilities, 29 (35%) only in NHS, and 8 only in private facilities.  Table 1 summarizes the final answers for the second Delphi round questionnaires. After the most important deficiencies were detected, an expert advisory panel used a new two-round Delphi method to describe some recommendations in order to reduce some of the problems detected.  Table 2 summarizes the final recommendations from the expert panel. The fifteen recommendations achieved an important consensus as all of them were ranged as “strongly agree” or “agree” by more than 80% of the members of the advisory panel.

## 4. Discussion

Although patients usually show a good adherence to individualized treatment strategies, the feasibility related to ophthalmologist and Healthcare resources, in addition to an increased number of patients with the need to be attended, carries a major clinical issue with the consequence of vision loss and suboptimal results compared to that achieved by the pivotal clinical trials [22]. Using modified Delphi methodology, a national panel of experts in nv-AMD treatment looked for potential deficiencies in real-life nv-AMD treatment and proposed 15 actions that may help to improve the outcomes of patients with nv-AMD. The level of agreement with the final recommendations among Spanish retinal specialist was high indicating that some actions can be applied in order to reduce disarrangements inpatient flow and nv-AMD management.

The results of this analysis showed that there was a high degree of agreement about the signs and symptoms to refer a patient to a retina specialist (Table 1). However, the total time spent in each visit might determine the total amount of patients that can be efficiently attended during every working day [23]. The extensive use of retina clinics in NHS facilities might explain that a routine fluorescein angiography is not always performed in all new cases and that an indocyanine green angiography is infrequently done in an attempt to reduce the time of the visit (Table 1). On the other hand, the three initial consecutive monthly intravitreal injections (loading phase) are almost always administered, as these may not be perceived as a significant burden assuming that they reduce the proportion of patients with vision loss [24]. However, during the loading phase scheduled visits, most retina specialists performed visual acuity (VA) exams and OCT imaging that might be avoided (Table 1). In addition, few Spanish ophthalmologists consider that VA and OCT should be always performed by technical staff, but instead by themselves (Table 1). These two points might lead to unnecessary diagnostic testing and lengthening of each visit. So to define in each center an efficient structure where technicians, optometrists and nurses have clear roles that allow a faster patient flow might be mandatory.

Current Spanish and European guidelines recommend a PRN dosing strategy after the loading dose, where injections are administered when visual acuity loss and signs of lesion activity occur [4–6, 17, 23]. This is probably the reason why the most valued protocol for the follow-up of nv-AMD in Spain turned out to be PRN (Table 1). However, this protocol requires monthly monitoring visits which are considered the biggest burden to deal with [23]. Time delays are frequent beyond the recommended interval, and this might usually result in preventable vision loss.

In order to lengthen the period between follow-up examinations, a protocol called* Inject and Extend* or* Treat and Extend Regimen* (TER) has also been described [25–27]. This approach involves continuous maintenance treatment, but at less than monthly intervals, with the dosing schedule determined by VA, clinical examination, and OCT findings [25–27]. However, TER is not often used in Spain (Table 1).

*The “wait and extend”* strategy is considered by the Spanish retina physicians the least appropriate regimen, although it is considered the most easily suitable in case of busy agendas, and consequently is frequently used in Spain (Table 1). With this protocol, follow-up visits are progressively spread out to a maximum of 8 weeks apart in the absence of visual acuity loss and signs of lesion activity, but without reinjections unless the OCT images evidence exudative changes. This regimen has shown to be efficient and safe in patients with neovascular AMD, reducing both the burden of the number of injections and follow-up visits [28]. However, other studies with a less strict protocol found that when the follow-up visits were gradually spaced out, the mean VA improvement was only 0.7 letters at 1 year [29]. This indicates that follow-up and treatment protocols in each hospital must be as strict as possible, and audits must be periodically performed to assure adherence. It is reasonable that strict protocols, as these followed in clinical trials, may help to improve results in real-life treatment of AMD patients.

The majority of retina specialists surveyed considered that the best option is to perform intravitreal injections on the same day of follow-up visit. However, they also believe that to perform the injection on the same day represents a bigger burden due to flow-chart organization reasons (Table 1). Another issue is that many of the Spanish retinal physicians prefer to inject in the operating room because it is safer [30] (Table 1). In order to perform an injection on the same day as the follow up visit, it is important to organize the clinic so as to reduce the time spent per injection as well as minimizing waiting times for appointments. Injecting on the same day as the follow-up visit might avoid scheduling unnecessary future appointments for the patient.

The treatment may be done in a clean room just as safely as in an operating room as is recommended by the SERV guidelines for intravitreal injections [31]. The most important aspects are the previous topical administration of povidone iodine, the use of sterile material and gloves, and an adequate injection technique, rather than the treatment environment. Finally, the injection may be performed by a trained ophthalmologist and it is not necessary that a retina specialist perform the procedure.

Recently, the identification of “nonresponders” or “poor responders” is relevant as these might require more visits and injections. There is not any clear defined criterion to identify such cases. In this Delphi study, the participant agreed that an absolute bad responder is a patient losing vision with anatomical worsening after antiangiogenic therapy. In this case the most frequent protocol identified in the survey is to perform an indocyanine green angiography in order to identify as the presence of polypoidal choroidal vasculopathy or retinal angiomatous proliferation [32, 33]. The most frequently employed second line treatment was combination therapy with photodynamic therapy and antiangiogenic drugs [34]. The second option was to swap the antiangiogenic drug [35] (Table 1). It is possible that the former option will increase in the future with the commercialization of new approved antiangiogenic drugs like aflibercept [35]. Most participants in this study agreed that the best moment to determine the degree of response of a patient to the initiated therapy is after finishing the loading phase [36].

Another important gap is that once a patient has been treated and followed in a hospital, hospital ophthalmologists seldom refer this patient to the general ophthalmologist although they have reached a low level of vision, or a disciform scar have developed (Table 1). This is probably because some studies like HORIZON and SEVEN-UP have shown that nv-AMD may remain active for a number of years [9, 37]. However, sometimes treatment may be discontinued in presence of signs active disease [38, 39]. For example, treatment may be deferred or terminated in situations where further visual improvement appears unlikely, for example, severe RPE tears involving the foveal center, or in the context of significant coexisting geographic atrophy or subfoveal scar formation [40]. In those cases the patient may be referred to a general ophthalmologist in an outpatient setting.

In summary, after the most important deficiencies were detected, an expert advisory panel used a new two-round Delphi method to describe some recommendations in order to reduce some of the problems detected.

Ideally, a personalized retreatment that keeps in mind the risks and benefits and avoid the over- and undertreatment of the patient should be done. Generally it is advisable to start the treatment with a loading phase of three injections. In order to reduce assistance burden, complementary examinations could be avoided during the loading phase or be reduced to a minimum, exploring only VA and OCT if the patient informs there is a problem. Moreover, in some monitoring visits, in which the treatment has been previously decided, the examinations could be omitted. It would be advisable that examinations such as VA and OCT would be performed by technical staff, while the ophthalmologist interprets the results and makes treatment decisions. When following the PRN protocol, reinjection is recommended in the case of evidence of disease activity in the OCT, new hemorrhage or vision loss due to the disease activity. The TER regimen could be a useful protocol to reduce the number of follow-up visits and the burden of the disease. In the presence of a relapse it is recommended to continue with the treatment until the retreatment criteria disappears. When retreating due to a relapse, if it is not possible to follow established monitoring guidelines, a new loading phase may be considered without performing the associated examinations. Ideally, the relapses should be treated the same day they are diagnosed. It is as adequate and safe to perform intravitreal injections in a clean room as in an operating room. Absolute nonresponders are defined as those who after the three loading doses still have worsening VA and OCT. In nonresponders, the treatment has to be decided after doing anindocyanine green angiography (to rule out diseases such as polypoidal choroidopathy or retina angiomatous proliferation). It would be recommendable to refer patients to a general ophthalmologist who have not presented with retreatment criteria in the last 12 months or who have the disease in a disciform state.

Although these are commonly occurring problems and some suggested solutions, the authors acknowledge that not all of these issues will apply to every clinic. Moreover, this Delphi study was done with Spanish retina specialists and the problems may not be the same in NHS from different countries. However, it is vital to detect the main bottlenecks in hospitals and clinical services to provide a fast and efficient service for patients with nv-AMD.

## Figures and Tables

**Table 1:** Final answers for the second Delphi round questionnaires.

Question	Almost never	Sometimes	Frequently	Almost always
Diagnosis and initial treatment				
(1) Do you consider the metamorphopsia a red flag symptom?	0%	2%	15%	83%
(2) Do you consider a strong decrease of VA a key symptom of alert?	0%	1%	22%	77%
(3) Do you consider the appearance of a central scotoma a key symptom of alert?	0%	1%	21%	78%
(4) Do you consider a macular hemorrhage in a patient with drusen a key symptom of alert?	0%	1%	5%	94%
(5) Do you consider the macular edema in a patient with drusen a key symptom of alert?	0%	0%	21%	79%
(6) Do you consider the corrected VA an essential initial test?	0%	1%	8%	91%
(7) Do you consider the PPBMC an essential initial test?	0%	4%	9%	87%
(8) Do you consider the macular OCT an essential initial test?	0%	0%	6%	94%
(9) Do you consider the FA an essential initial test?	0%	18%	42%	40%
(10) Do you consider the loading doses (3 intravitreal injections) the routine way to begin treatment in all cases?	2%	7%	21%	70%
(11) Do you consider the initial loading dose feasible from a socio-sanitary point of view?	5%	15%	46%	34%
(12) Do you consider the PRN regimen (1 intravitreal injection + PRN) the routine way to start treatment in all the cases?	35%	40%	18%	7%
(13) Do you consider a complete series of diagnostic tests necessary during the loading phase?	25%	49%	12%	14%
(14) Do you consider a limited amount of diagnostic testing necessary during the loading phase?	7%	39%	20%	34%
(15) Do you consider monthly complete examinations in the PRN regimen feasible in NHS hospitals (Consider “complete examination” VA, OCT PPBMC)?	35%	48%	15%	2%
(16) Do you consider monthly complete examinations in the PRN regimen feasible in private hospitals (Consider “complete examination” VA, OCT PPBMC)?	8%	27%	45%	20%

Individualized treatment therapy				
(17) Do you have in consideration the balance of risk/profit in deciding which guidelines to follow?	1%	6%	57%	36%
(18) Do you consider monthly treatment the most suitable regimen?	16%	21%	40%	23%
(19) Do you consider monthly treatment feasible in NHS hospitals?	73%	22%	3%	2%
(20) Do you consider monthly treatment feasible in public hospitals?	30%	37%	28%	5%
(21) Do you consider the PRN regimen with monthly visits the most suitable practice?	2%	25%	51%	22%
(22) Do you consider the PRN regimen with monthly visits feasible in NHS hospitals?	32%	36%	30%	2%
(23) Do you consider the PRN regimen with monthly visits feasible in private hospitals?	8%	24%	60%	8%
(24) Do you consider the T&E regimen the most suitable practice?	3%	53%	38%	6%
(25) Do you consider the T&E regimen feasible in NHS hospitals?	8%	38%	52%	2%
(26) Do you consider the T&E regimen feasible in private hospitals?	5%	25%	58%	12%
(27) Do you consider the W&E regimen the most suitable practice?	12%	56%	31%	1%
(28) Do you consider the W&E regimen feasible in NHS hospitals?	2%	26%	67%	5%
(29) Do you consider the W&E regimen feasible in private hospitals?	4%	19%	69%	8%
(30) Do you consider that, in general, most of the patients will be properly treated with seven injections during the first year of the treatment?	0%	10%	80%	10%
(31) Do you consider that, in general, most of the patients will be properly treated with four injections during the second year of the treatment?	1%	27%	52%	5%
(32) Do you consider it suitable to perform the intravitreal injection on the same day of the follow-up visit?	3%	23%	22%	52%
(33) Do you consider it feasible to perform the intravitreal injection on the same day of the follow-up visit in NHS hospitals?	29%	43%	14%	14%
(34) Do you consider it feasible to perform the intravitreal injection on the same day of the follow-up visit in private hospitals?	5%	24%	42%	29%
(35) Aside from the logistical factors, if you have a clean room in the consulting area, would you consider it appropriate to perform an intravitreal injection there?	38%	19%	19%	24%
(36) Do you consider performing the intravitreal injection in a clean room as safe as in the operating room?	13%	22%	32%	32%
(37) Do you consider it necessary that the retinal specialist perform himself VA check?	25%	42%	20%	13%
(38) Do you consider it necessary that the retinal specialist perform himself the OCT?	15%	32%	27%	26%
(39) Do you consider it necessary that the retinal specialist perform himself the FA?	12%	20%	27%	41%
(40) Do you consider it necessary that the retinal specialist perform by himself the PPBMC?	2%	16%	24%	58%

Nonresponders and referral to general ophthalmologist				
(41) Do you consider an absolute nonresponder a patient with worsening VA and OCT macular thickness post-treatment?	0%	1%	44%	55%
(42) In the case of a nonresponder, should the checkup interval be reduced to 15 days since the last injection in order to test for a response?	18%	48%	19%	15%
(43) In the case of a nonresponder, should the AGF and ICG be repeated to rule out pathologies such as CP or RAP?	0%	13%	28%	59%
(44) Do you consider visual acuity less than 20/400 a criteria to refer the patient to the GO?	13%	59%	22%	6%
(45) Do you consider visual acuity less than 20/200 a criteria to refer the patient to the GO?	42%	49%	6%	3%
(46) Do you consider a fibrosis over 50% of the lesion (disciform scar) criteria to refer the patient to the GO?	7%	48%	38%	7%
(47) Do you consider the absence of retreatment criteria during the last 6 months a criterion to refer the patient to the GO?	37%	48%	15%	0%
(48) Do you consider the absence of retreatment criteria during the last 9 months a criterion to refer the patient to the GO?	25%	34%	35%	6%
(49) Do you consider the absence of retreatment criteria during the last 12 months a criterion to refer the patient to the GO?	13%	31%	33%	23%
(50) How many monthly injections do you usually perform to treat the relapses?	At least, a fixed load phase until the criteria of re-treatment disappeared 47%	Only until the criteria of retreatment disappeared 53%		
(51) After how many injections do you define a nonresponder?	First injection 16%	After the loading phase 60%	After six months of treatment 20%	After first year of treatment 4%
(52) In the case of a nonresponder the second line treatment should be to	Interrupt the treatment with anti-VEGF 11%	Change the treatment with other anti-VEGF 42%	Use combined therapy 47%	Use the same treatment 0%

VA: best corrected visual acuity; OCT: optimal coherence tomography; PPBMC: posterior pole biomicroscopy; FA: fluorescein angiography; NHS: National Health System; PRN: pro re nata; T&E: treat and extend; W&E: wait and extend; ICG: indocyanine green angiography; CP: choroidal vascular polidopipathy; RAP: retinal angiomatous proliferation; GO: general ophthalmologist.

**Table 2:** Final recommendations.

Item	Percentage of strongly agree	Percentage of agree	Percentage of neither agree nor disagree	Percentage of disagree	Percentage of strongly disagree
The results of antiangiogenic therapy could differ from the results of clinical trials because these protocols are frequently difficult to use in real clinical practice.	62,5	37,5	0	0	0

Ideally, a personalized retreatment that keeps in mind the risks and benefits and avoids the over- and undertreatment of the patient should be done.	75	25	0	0	0

Generally it is advisable to begin the treatment with a loading phase of three injections.	87,5	12,5	0	0	0

In order to reduce assistance burden, complementary examinations could be avoided during the loading phase or be reduced to a minimum exploring only VA and OCT.	56,25	31,25	12,5	0	0

In some monitoring visits in which the treatment has been previously decided, the examinations could be omitted.	43,75	37,5	12,5	0	6,25

It would be advisable that examinations such as VA and OCT would be performed by technical staff, while the ophthalmologist interprets the results and makes treatment decisions	50	43,75	6,25	0	0

In case of following the PRN regimen, reinjection is recommended in the case of evidence of disease activity in the OCT, new hemorrhage or vision loss due to the disease activity.	62,5	37,5	0	0	0

The T&E regimen could be a useful protocol to reduce the number of revisions and the burden of the disease.	37,5	56,25	0	6,25	0

In the presence of a relapse it is recommended to continue with the treatment until the retreatment criteria disappears.	43,25	50	6,25	0	0

When retreating due to a relapse, if it is not possible to follow established monitoring guidelines, a new loading phase may be considered without performing the associated examinations	37,5	56,25	6,25	0	0

Ideally, the relapses should be treated the same day they are diagnosed.	62,25	25	12,5	0	0

It is as adequate and safe to perform intravitreal injections in a clean room as in an operating room.	56,25	31,25	12,5	0	0

“Absolute” nonresponders are defined as those who after the three loading doses still have worsening VA and OCT	68,75	31,25	0	0	0

In nonresponders, the guidelines have to be decided after doing an ICG (to rule out diseases as CP or RAP)	62,5	31,25	6,25	0	0

It would be recommendable to refer patients to a general ophthalmologist who have not presented with retreatment criteria in the last 12 months or who have the disease in a disciform state.	50	31,25	12,5	6,25	0

## References

[1] Soubrane G., Cruess A., Lotery A. (2007). Burden and health care resource utilization in neovascular age-related macular degeneration: findings of a multicountry study. *Archives of Ophthalmology*.

[2] Rosenfeld P. J., Brown D. M., Heier J. S., Boyer D. S., Kaiser P. K., Chung C. Y., Kim R. Y. (2006). Ranibizumab for neovascular age-related macular degeneration. *The New England Journal of Medicine*.

[3] Schmidt-Erfurth U., Kaiser P. K., Korobelnik J.-F., Brown D. M., Chong V., Nguyen Q. D., Ho A. C., Ogura Y., Simader C., Jaffe G. J., Slakter J. S., Yancopoulos G. D., Stahl N., Vitti R., Berliner A. J., Soo Y., Anderesi M., Sowade O., Zeitz O., Norenberg C., Sandbrink R., Heier J. S. (2014). Intravitreal aflibercept injection for neovascular age-related macular degeneration: Ninety-six-week results of the VIEW studies. *Ophthalmology*.

[4] Lalwani G. A., Rosenfeld P. J., Fung A. E., Dubovy S. R., Michels S., Feuer W., Davis J. L., Flynn H. W., Esquiabro M. (2009). A variable-dosing regimen with intravitrealranibizumab for neovascular age-related macular degeneration: year 2 of the PrONTO Study. *The American Journal of Ophthalmology*.

[5] Martin D. F., Maguire M. G., Ying G.-S., Grunwald J. E., Fine S. L., Jaffe G. J. (2011). Ranibizumab and bevacizumab for neovascular age-related macular degeneration. *The New England Journal of Medicine*.

[6] Busbee B. G., Ho A. C., Brown D. M., Heier J. S., Suñer I. J., Li Z., Rubio R. G., Lai P. (2013). Twelve-month efficacy and safety of 0.5 mg or 2.0 mg ranibizumab in patients with subfoveal neovascular age-related macular degeneration. *Ophthalmology*.

[7] Hernandez-Pastor L. J., Ortega A., García-Layana A., Giráldez J. (2010). Cost-effectiveness of ranibizumab compared with pegaptanib in neovascular age-related macular degeneration. *Graefe's Archive for Clinical and Experimental Ophthalmology*.

[8] Krüger Falk M., Kemp H., Sorensen T. L. (2013). Four-year treatment results of neovascular age-related macular degeneration with ranibizumab and causes for discontinuation of treatment. *American Journal of Ophthalmology*.

[9] Singer M. A., Awh C. C., Sadda S., Freeman W. R., Antoszyk A. N., Wong P., Tuomi L. (2012). HORIZON: an open-label extension trial of ranibizumab for choroidal neovascularization secondary to age-related macular degeneration. *Ophthalmology*.

[10] Tano Y., Ohji M., EXTEND-I Study Group (2011). Long-term efficacy and safety of ranibizumab administered pro re nata in Japanese patients with neovascular age-related macular degeneration in the EXTEND-I study. *Acta Ophthalmologica Scandinavica*.

[11] Querques G., Azrya S., Martinelli D., Berboucha E., Feldman A., Pece A., Coscas G., Soubrane G., Souied E. H. (2010). Ranibizumab for exudative age-related macular degeneration: 24-month outcomes from a single-centre institutional setting. *British Journal of Ophthalmology*.

[12] Biarnés M., Monés J., Villalbí J. R., Arias L. (2011). As-needed treatment with ranibizumab 0.5 mg in patients with neovascular age-related macular degeneration. *European Journal of Ophthalmology*.

[13] Lees N. M., Lievaart J. J. (2013). Expert opinion on ranking risk factors for subclinical mastitis using a modified Delphi technique. *New Zealand Veterinary Journal*.

[14] Hasson F., Keeney S., McKenna H. (2000). Research guidelines for the Delphi survey technique. *Journal of Advanced Nursing*.

[15] Keeney S., Hasson F., McKenna H. (2006). Consulting the oracle: ten lessons from using the Delphi technique in nursing research. *Journal of Advanced Nursing*.

[16] Tolsgaard M. G., Todsen T., Sorensen J. L., Ringsted C., Lorentzen T., Ottesen B., Tabor A. (2013). International multispecialty consensus on how to evaluate ultrasound competence: a Delphi consensus survey. *PLoS ONE*.

[17] Ruiz-Moreno J. M., Arias-Barquet L., Armadá-Maresca F., Boixadera-Espax A., García-Layana A., Gómez-Ulla-De-Irazazábal F., Monés-Carilla J., Piñerobustamante A., Suárez-De-Figueroa M. (2009). Guidelines of clinical practice of the SERV: treatment of exudative age-related macular degeneration (AMD). *Archivos de la Sociedad Española de Oftalmología*.

[18] Fink A., Kosecoff J., Chassin M., Brook R. H. (1984). Consensus methods: characteristics and guidelines for use. *The American Journal of Public Health*.

[19] Singh P., Aggarwal R., Zevin B., Grantcharov T., Darzi A. (2014). A global Delphi consensus study on defining and measuring quality in surgical training. *Journal of The American College of Surgeons*.

[20] Zevin B., Bonrath E. M., Aggarwal R., Dedy N. J., Ahmed N., Grantcharov T. P. (2013). Development, feasibility, validity, and reliability of a scale for objective assessment of operative performance in laparoscopic gastric bypass surgery. *Journal of the American College of Surgeons*.

[21] Nagpal K., Arora S., Abboudi M., Vats A., Wong H. W., Manchanda C., Vincent C., Moorthy K. (2010). Postoperative handover: problems, pitfalls, and prevention of error. *Annals of Surgery*.

[22] Mitchell P., Korobelnik J.-F., Lanzetta P., Holz F. G., Prünte C., Schmidt-Erfurth U., Tano Y., Wolf S. (2010). Ranibizumab (Lucentis) in neovascular age-related macular degeneration: evidence from clinical trials. *British Journal of Ophthalmology*.

[23] Casaroli-Marano R., Roura M. (2013). Availability of resources for patients with wet age-related macular degeneration. Optimal study. *Archivos de la Sociedad Española de Oftalmología*.

[24] Menon G., Chandran M., Sivaprasad S., Chavan R., Narendran N., Yang Y. (2013). Is it necessary to use three mandatory loading doses when commencing therapy for neovascular age-related macular degeneration using bevacizumab? (BeMOc Trial). *Eye (Basingstoke)*.

[25] Spaide R. F. (2009). The as-needed treatment strategy for choroidal neovascularization: a feedback-based treatment system. *American Journal of Ophthalmology*.

[26] Toalster N., Russell M., Ng P. (2013). A 12-month prospective trial of inject and extend regimen for ranibizumab treatment of age-related macular degeneration. *Retina*.

[27] Oubraham H., Cohen S. Y., Samimi S., Marotte D., Bouzaher I., Bonicel P., Fajnkuchen F., Tadayoni R. (2011). Inject and extend dosing versus dosing as needed: a comparative retrospective study of ranibizumab in exudative age-related macular degeneration. *Retina*.

[28] Arias L., Roman I., Masuet-Aumatell C. (2011). One-year results of a flexible regimen with ranibizumab therapy in macular degeneration: relationship with the number of injections. *Retina*.

[29] Cohen S. Y., Dubois L., Tadayoni R., Fajnkuchen F., Nghiem-Buffet S., Delahaye-Mazza C., Guiberteau B., Quentel G. (2009). Results of one-year's treatment with ranibizumab for exudative age-related macular degeneration in a clinical setting. *American Journal of Ophthalmology*.

[30] Casparis H., Wolfensberger T. J., Becker M. (2014). Incidence of presumed endophthalmitis after intravitreal injection performed in the operating room: a retrospective multicenter study. *Retina*.

[31] Gómez-Ulla F., Basauri E., Arias L., Martínez-Sanz F. (2009). Management of intravitreal injections. *Archivos de la Sociedad Espanola de Oftalmologia*.

[32] Stangos A. N., Gandhi J. S., Nair-Sahni J., Heimann H., Pournaras C. J., Harding S. P. (2010). Polypoidal choroidal vasculopathy masquerading as neovascular age-related macular degeneration refractory to ranibizumab. *American Journal of Ophthalmology*.

[33] Talks J., Koshy Z., Chatzinikolas K. (2007). Use of optical coherence tomography, fluorescein angiography and indocyanine green angiography in a screening clinic for wet age-related macular degeneration. *The British Journal of Ophthalmology*.

[34] Tozer K., Roller A. B., Chong L. P., Sadda S., Folk J. C., Mahajan V. B., Russell S. R., Boldt H. C., Sohn E. H. (2013). Combination therapy for neovascular age-related macular degeneration refractory to anti-vascular endothelial growth factor agents. *Ophthalmology*.

[35] Ho V. Y., Yeh S., Olsen T. W., Bergstrom C. S., Yan J., Cribbs B. E., Hubbard G. B. (2013). Short-term outcomes of aflibercept for neovascular age-related macular degeneration in eyes previously treated with other vascular endothelial growth factor inhibitors. *American Journal of Ophthalmology*.

[36] Krebs I., Glittenberg C., Ansari-Shahrezaei S., Hagen S., Steiner I., Binder S. (2013). Non-responders to treatment with antagonists of vascular endothelial growth factor in age-related macular degeneration. *British Journal of Ophthalmology*.

[37] Rofagha S., Bhisitkul R. B., Boyer D. S., Sadda S. R., Zhang K. (2013). Seven-year outcomes in ranibizumab-treated patients in ANCHOR, MARINA, and HORIZON: a multicenter cohort study (SEVEN-UP). *Ophthalmology*.

[38] Amoaku W. (2011). Ranibizumab: the clinician's guide to commencing, continuing, and discontinuing treatment. *Eye*.

[39] Gaudric A., Cohen S. Y. (2010). When should anti-vascular endothelial growth factor treatment be stopped in age-related macular degeneration?. *American Journal of Ophthalmology*.

[40] Keane P. A., Tufail A., Patel P. J. (2011). Management of neovascular age-related macular degeneration in clinical practice: initiation, maintenance, and discontinuation of therapy. *Journal of Ophthalmology*.

